# Analysis of risk factors and intervention measures for postoperative bleeding in patients with diabetes after proctologic surgery based on a logistic regression model

**DOI:** 10.3389/fmed.2025.1559707

**Published:** 2025-07-22

**Authors:** Qiuxiang Yu, Peiyao Li, Dan Li, Lin Wang, Guoshuang Yu, Rui Li

**Affiliations:** ^1^Department of Proctology, China-Japan Friendship Hospital, Beijing, China; ^2^Department of International Coloproctology, China-Japan Friendship Hospital, Beijing, China; ^3^Department of International Ward 3, China-Japan Friendship Hospital, Beijing, China

**Keywords:** proctologic operation, postoperative hemorrhage, diabetes, risk factors, proctologic surgery

## Abstract

**Objective:**

The purpose of this study was to analyse the risk factors for bleeding after proctologic surgery, provide a reference for clinical management and improve postoperative recovery in patients.

**Methods:**

This study retrospectively analyzed the clinical data of patients undergoing proctologic surgery in our hospital. Patients with postoperative bleeding comprised the study group, whereas those without bleeding formed the control group. A logistic regression model was used to analyse the risk factors for postoperative bleeding in patients undergoing proctologic surgery. A *p*-value < 0.05 was considered statistically significant.

**Results:**

The study found that the risk of postoperative bleeding in men was significantly higher than in women (*p* < 0.001). Hypertension was identified as an independent risk factor for bleeding (*p* < 0.001). The incidence of bleeding was significantly higher in patients who received preoperative antibiotics (*p* = 0.032). The proportion of patients with postoperative dry stool in the bleeding group was significantly higher than in the control group (*p* < 0.001). The under the curve (AUC) of the prediction model was 0.820, 95%CI: 0.760–0.880, the sensitivity was 72.4%, and the specificity was 82.7%.

**Conclusion:**

This study demonstrated that the risk of postoperative bleeding after proctologic surgery was closely associated with several factors, including male sex, hypertension, preoperative antibiotic use and postoperative constipation, all of which significantly increased the risk of bleeding.

## 1 Introduction

Proctologic diseases cover a variety of conditions in the proctologic region, usually manifested as haematochezia, mass prolapse, proctologic pain and swelling (proctologic pain and swelling, for reference), perianal ulceration and pus. The occurrence of proctologic diseases is related to many factors, including defecation habits, eating habits, personal hygiene and overall health status (1). Proctologic surgery is a common and effective method for the treatment of these diseases but is also associated with a high risk of complications, especially postoperative bleeding (2).

According to the International Diabetes Federation, the number of people with diabetes worldwide has reached hundreds of millions and is expected to continue to grow (3). In the treatment of proctologic diseases, the surgical needs of patients with diabetes are increasing (4). The coexistence of proctologic diseases and diabetes not only seriously affects the quality of life of patients but also places a heavy burden on the medical system (5).

Studies have shown that the risk of postoperative bleeding in patients with proctologic diseases combined with diabetes is significantly higher than in patients without diabetes (6–8). This phenomenon is related to the effects of diabetes on vascular health, immune function and healing ability (9). Postoperative bleeding is a common and serious complication in surgery, especially in proctologic procedures. Bleeding may be associated with a variety of factors, including the patient’s underlying condition, preoperative evaluation, surgical techniques and postoperative care (10).

Due to poor blood glucose control, patients with diabetes may experience vascular dysfunction, reduced immunity and impaired healing ability, all of which may increase the risk of postoperative bleeding (11). Therefore, identifying and managing the risk factors for bleeding after proctologic surgery in patients with diabetes is essential to reduce the incidence of bleeding and improve patient prognosis (11, 12). Strengthening preoperative evaluation and postoperative nursing can substantially enhance the safety and therapeutic outcomes of proctologic surgery in patients with diabetes.

The purpose of this study was to explore the risk factors for bleeding after proctologic surgery in patients with diabetes through logistic regression analysis. Based on the findings, effective interventions were proposed to reduce the risk of postoperative bleeding and improve the quality of recovery. This study provides a reference for clinical practice to help doctors conduct risk assessments before surgery and formulate individualized management plans. These measures aim to improve postoperative safety, reduce complication rates, optimize medical resource use, alleviate the healthcare burden and ultimately enhance patients’ quality of life and recovery. This study aims to offer new perspectives for the management of proctologic surgery in patients with diabetes and support the further development of clinical practice.

## 2 Materials and methods

### 2.1 General information

This study used a retrospective study design to collect data on patients who underwent proctologic surgery in our hospital between March 2019 and March 2023, totalling 185 cases. According to the presence of postoperative bleeding, the patients were divided into two groups: the study group included 87 patients with postoperative bleeding, and the control group included 98 patients without postoperative bleeding.

The inclusion criteria included the following: (1) All patients should be patients undergoing proctologic surgery. (2) Patients needed to be diagnosed with diabetes and provide a clear medical history record at admission. Types of diabetes included type 1 diabetes and type 2 diabetes. (3) The type of surgery should be common proctologic surgery, such as the Milligan–Morgan procedure, Hemorrhoidectomy, anal fistulotomy or anal fistula seton procedure. (4) Patients should be followed up for at least 14 days after surgery to accurately record postoperative bleeding. (5) All patients had signed informed consent before admission, allowing the use of their medical data for research.

The exclusion criteria included the following: (1) Patients who needed first aid due to acute diseases complicated by diabetes (such as diabetic ketoacidosis or uraemia). (2) Patients with severe heart disease, chronic liver dysfunction or severe renal insufficiency, which may affect the operation or postoperative recovery. (3) Patients with complex proctologic reconstruction surgery or other major surgery. (4) Patients who failed to follow up for at least 14 days after surgery and whose postoperative bleeding could not be accurately recorded.

This study was approved by the Hospital Ethics Committee under approval numbers 2019-30-K24 and 2021-101-K62.

### 2.2 Data source

The research data mainly came from the patients’ electronic medical records, surgical records and postoperative follow-up records. Through the hospital’s electronic health record system, the clinical information of the relevant patients was extracted.

### 2.3 Data collection

This included the patient’s age, gender and body mass index (BMI). The presence of hypertension and the type of diabetes (type 1 or type 2) were recorded. Surgical methods, operation time and preoperative use of antibiotics were also noted. Postoperative variables included defecation status (dry or unobstructed), postoperative bleeding and postoperative hospital stay. Data on preoperative prothrombin time (PT), activated partial thromboplastin time (APTT), hemoglobin, platelet count, fasting blood glucose and other indicators were also collected.

### 2.4 Postoperative care

The vital signs of the patients were monitored regularly after the operation, including body temperature, heart rate, respiratory rate and blood pressure, to promptly detect abnormalities. According to the patient’s needs and the degree of pain, appropriate analgesic drugs were administered to ensure patient comfort and promote recovery. The patient’s bleeding was observed and followed up within 14 days after surgery. The wound and defecation were regularly checked, and any bleeding was recorded promptly to ensure timely treatment. In this study, postoperative bleeding refers to the occurrence of blood drops or spotting after defecation. Patients were guided to keep the wound dry and clean and avoid excessive friction. The wound dressing was replaced when necessary, wound healing was observed and infection was prevented. According to the patient’s postoperative recovery, diet was gradually adjusted. In the early stage, a liquid diet could be selected, followed by a gradual transition to soft food, avoiding spicy and irritating food to promote intestinal function recovery. Patients were encouraged to maintain appropriate defecation patterns. If necessary, soft stools could be used to help relieve constipation and avoid re-injury of the wound. Blood glucose levels were regularly monitored, and the amount of insulin or oral drugs was adjusted to ensure that blood glucose was controlled within a reasonable range and to promote postoperative wound healing.

### 2.5 Statistical methods

Statistical analysis was conducted using SPSS 26.0 software. Normality was assessed using the Kolmogorov–Smirnov test. Data conforming to normality were expressed as mean ± standard deviation and compared using the independent samples *t*-test. Non-normally distributed data were presented as the median (interquartile range) and analyzed using the Mann–Whitney U test. Count data were expressed as frequency (n) or rate (%). The *χ*^2^ test was used for those who met the conditions, and Fisher’s exact probability method was used for those who did not. Logistic regression analysis was used to analyse the suspicious risk factors for postoperative bleeding. The prediction model was further verified by the receiver operator characteristic curve (ROC). *p* < 0.05 was considered statistically significant.

## 3 Results

### 3.1 Univariate analysis

This study analyzed the single-factor effect of bleeding-related variables in 185 patients undergoing proctologic surgery, comparing different characteristics. According to the presence of postoperative bleeding, patients were divided into a control group (no bleeding, *n* = 98) and a bleeding group (bleeding, *n* = 87). The average age of the control group was 44.71 ± 7.47 years and that of the bleeding group was 45.89 ± 11.17 years.

Univariate analysis showed that, in terms of gender distribution, men accounted for a higher proportion in the bleeding group, with 51 men in the control group and 64 in the bleeding group. This difference was statistically significant (*χ*^2^ = 9.076, *p* = 0.003). The number of patients with hypertension in the bleeding group was also significantly higher than in the control group, with 9 in the control group and 35 in the bleeding group (*χ*^2^ = 20.505, *p* < 0.001). The proportion of patients with dry and hard defecation in the bleeding group was higher, with 15 cases in the control group and 41 in the bleeding group (*χ*^2^ = 22.108, *p* < 0.001). In terms of preoperative antibiotic use, the usage rate in the bleeding group was significantly higher than that in the control group (*χ*^2^ = 4.581, *p* = 0.032).

Univariate analysis indicated that male gender, hypertension, dry and hard postoperative defecation and preoperative antibiotic use were significantly associated with an increased risk of bleeding. There were no significant differences between the two groups in terms of BMI, type of diabetes, smoking, drinking, surgical method, operation time, PT, APTT, platelet count or fasting blood glucose (see Table 1).

**Table 1:** Univariate analysis of influencing factors of postoperative bleeding after proctologic surgery.

Item	Control group (*n* = 98)	Bleeding group (*n* = 87)	*t*/*χ*^2^	*p*
Mean age (year)	44.71 ± 7.47	45.89 ± 11.17	−0.847	0.398
Gender			9.076	0.003
Male	51	64		
Female	47	23		
BMI index (kg/m^2^)	25.24 ± 2.18	25.54 ± 0.89	−1.168	0.244
Hypertension			20.505	<0.001
Yes	9	35		
No	89	52		
Type of diabetes			1.494	0.222
Type 1	55	41		
Type 2	43	46		
Postoperative defecation			22.108	<0.001
Hard drying	15	41		
Fluency	83	46		
Smoke			0.195	0.659
Yes	38	31		
No	60	56		
Drink			0.410	0.522
Yes	54	52		
No	44	35		
Surgical approaches			0.534	0.465
Tradition	7	4		
Microtrauma	91	83		
Preoperative antibiotics			4.581	0.032
Yes	42	51		
No	56	36		
Operation time	0.50 ± 0.44	0.48 ± 0.12	0.307	0.759
PT (s)	11.49 ± 0.91	11.63 ± 0.90	−1.065	0.288
APTT(s)	33.56 ± 1.73	33.55 ± 1.86	0.036	0.971
Hemoglobin (g/L)	137.07 ± 11.62	131.71 ± 12.92	2.966	0.003
Platelet count (10^9^/L)	248.91 ± 46.96	243.67 ± 47.35	0.755	0.451
Fasting blood glucose (g/L)	5.55 ± 0.57	5.63 ± 0.57	−1.061	0.290

### 3.2 Multivariate logistic analysis

Relevant variables that were statistically significant in the univariate analysis were included and assigned. Postoperative bleeding was used as the dependent variable. Gender (0 = man, 1 = woman), hypertension (0 = no, 1 = yes), preoperative antibiotic use (0 = no, 1 = yes), hemoglobin (actual value) and postoperative defecation (0 = dry and hard, 1 = unobstructed) were used as independent variables to further analyse the independent risk factors for postoperative bleeding.

**Figure 1:**
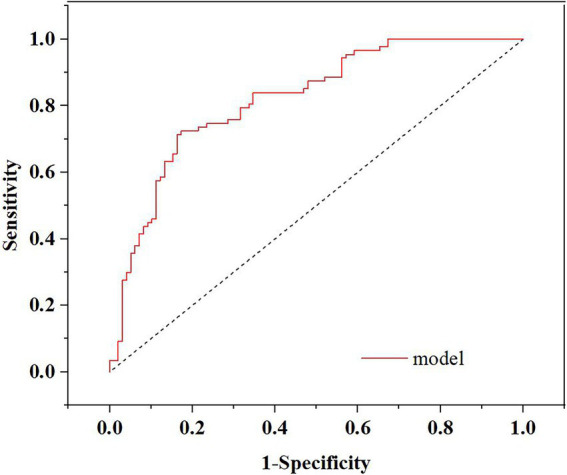
The ROC curve of the prediction model.

Multivariate logistic regression analysis showed that gender (*p* = 0.027, odds ratio [OR] = 2.435, 95% confidence interval [CI]: 1.108–5.352), hypertension (*p* < 0.001, OR = 7.277, 95% CI: 2.691–19.679) and postoperative defecation (*p* < 0.001, OR = 5.340, 95% CI: 2.252–12.664) were independent influencing factors for postoperative bleeding in patients with diabetes undergoing proctologic surgery (see Table 2). The prediction model was further verified by the ROC curve. The results showed that the area under the curve (AUC) of the prediction model was 0.820, 95%CI: 0.760–0.880, the sensitivity was 72.4%, and the specificity was 82.7%, as shown in Figure 1.

**Table 2:** Multivariate logistic analysis of postoperative bleeding after proctologic surgery.

Factors	B value	Standard error	Wald χ^2^ value	*p* value	OR value	95% confidence interval	
						Lower limit	Upper limit
Gender	0.890	0.402	4.909	0.027	2.435	1.108	5.352
Hypertension	1.985	0.508	15.287	0.000	7.277	2.691	19.679
Preoperative antibiotics	0.343	0.387	0.786	0.375	1.409	0.660	3.009
Postoperative defecation	1.675	0.441	14.460	0.000	5.340	2.252	12.664

## 4 Discussion

Proctologic surgery refers to surgery for anal and rectal diseases. Common operations include haemorrhoid resection, anal fistula incision, anal fissure resection and rectal cancer resection (13). This type of surgery is generally performed to treat patients with pain, bleeding, infection and other symptoms caused by various conditions. Although proctologic surgery is well established, it still carries the risk of postoperative complications such as bleeding and infection. Therefore, postoperative nursing and management are crucial for the recovery of patients. Diabetes is a metabolic disease characterized by long-term hyperglycaemia (14, 15). Patients with diabetes often have poor wound-healing ability after surgery due to their own metabolic disorders. It is worth noting that hyperglycaemia can impair vascular function, leading to microvascular disease, which in turn affects blood supply and nutrient transport, resulting in prolonged postoperative recovery (16). In addition, patients with diabetes often experience a decline in immune function, increasing the risk of postoperative infection.

This study found that the proportion of men in the postoperative bleeding group was significantly higher than that of women. This result is consistent with previous studies, suggesting that men may be more prone to bleeding-related complications in terms of physiology and behavior (17). From a physiological point of view, the anal canal of men is longer than that of women, so the wound length in men is usually greater during surgery. In addition, men often have poor compliance and may be more active than women after surgery, which increases the shear force on the wound, leading to increased trauma and a higher risk of bleeding. These factors may explain the high risk of postoperative bleeding in men.

The results of this study showed that hypertension was an independent risk factor for bleeding after proctologic surgery (*p* < 0.001). This finding is consistent with previous research. Hypertension is considered an important factor contributing to an increased risk of postoperative bleeding. Possible mechanisms include long-term damage to the vascular wall caused by hypertension, such as endothelial dysfunction and thickening of the vascular wall. These changes may make blood vessels more fragile and prone to rupture and bleeding. Persistent hypertension may increase the risk of postoperative bleeding, as it may lead to re-rupture of incompletely healed blood vessels (18).

The results of this study suggest a significant association between preoperative antibiotic use and the increased incidence of postoperative bleeding. This association may be influenced by the specific properties of different antibiotics, which can interfere with coagulation mechanisms or the intestinal flora. Certain antibiotics, particularly some cephalosporins such as cefoperazone and latamoxef, contain N-methyl sulfide tetrazolium side chains. These antibiotics can interact with vitamin K, inhibit the carboxylation of glutamic acid and subsequently produce abnormal thrombin, leading to coagulation disorders and increased bleeding risk. This effect is particularly concerning during the perioperative period, as disrupted coagulation mechanisms may result in excessive bleeding.

Additionally, disruption of the intestinal flora due to antibiotic use may contribute to an altered coagulation response. The intestinal microbiome is crucial for the synthesis of certain vitamins, such as vitamin K, which is essential for the normal coagulation cascade. Antibiotics that alter the balance of intestinal flora may reduce the bioavailability of vitamin K, exacerbating coagulation disorders and increasing the risk of bleeding (19).

Regarding the potential for ‘indication-related bias’, we acknowledge that patients with more severe or complex conditions may be more likely to receive antibiotics as part of their treatment plan, potentially confounding the observed relationship between antibiotic use and bleeding risk. This potential confounding factor should be considered when interpreting the results, as the underlying condition requiring antibiotic use may also contribute to the outcomes observed.

Postoperative constipation plays a substantial role in increasing the risk of bleeding after proctologic surgery. This study found that patients in the bleeding group had a higher incidence of dry stools than the control group, indicating a strong link between constipation and bleeding. Hard stools exert pressure on the surgical wound, which can cause physical trauma and hinder healing. Additionally, straining during defecation increases intra-abdominal pressure, which may compromise the integrity of blood vessels in the area, leading to rupture and bleeding. Constipation can also exacerbate local inflammation, further delaying wound recovery and increasing the risk of complications such as infection and pain (20).

To mitigate these risks, effective interventions should focus on softening stools and promoting regular bowel movements. Stool softeners, such as docusate sodium or polyethylene glycol, can reduce stool hardness and minimize strain during defecation. Increasing dietary fiber intake and maintaining adequate hydration also help to soften stools, reducing the risk of trauma to the wound. Early mobilization and abdominal massage can stimulate gut motility and relieve constipation. In more severe cases, enemas or suppositories may be used to facilitate bowel movements under medical supervision. Thus, incorporating early interventions to manage constipation is crucial for enhancing recovery and reducing complications in postoperative care for patients undergoing proctologic surgery.

This study has several limitations that should be acknowledged. The retrospective design may introduce information bias in data collection and outcome assessment, particularly in the documentation of bleeding events. The single-center nature and limited sample size may affect the generalisability of our findings to broader populations. Although we controlled for key confounding variables, other unmeasured factors, such as detailed antibiotic regimens or surgical techniques, could potentially influence the results. The relatively short follow-up period may also limit our ability to assess delayed bleeding complications. These limitations highlight the need for future multicentre prospective studies with larger sample sizes and standardized bleeding assessment protocols to validate our findings. Randomized controlled trials would be particularly valuable in investigating targeted interventions for the identified risk factors, such as optimized antibiotic selection or intensive blood pressure management in high-risk patients.

## 5 Conclusion

This study explored the influencing factors of postoperative bleeding in patients with diabetes undergoing proctologic surgery. The results showed that the risk of bleeding in men was significantly higher than that in women. Hypertension was identified as an independent risk factor for postoperative bleeding. Preoperative use of antibiotics was also significantly associated with the incidence of bleeding. In addition, postoperative constipation, especially dry and hard defecation, was associated with bleeding events and increased bleeding risk. Therefore, postoperative care should consider the patient’s sex, comorbidities, constipation management and hemoglobin level monitoring to reduce bleeding and improve recovery.

## Data Availability

The original contributions presented in the study are included in the article/supplementary material, further inquiries can be directed to the corresponding authors.
